# “Cigarettes led me back to smoking tik”: lived experience perspectives on tobacco use during substance use treatment and recovery in South Africa

**DOI:** 10.1186/s13722-026-00660-8

**Published:** 2026-03-12

**Authors:** Lorraine Samba Chiseya, Bronwyn Myers

**Affiliations:** 1 Division of Addiction Psychiatry, Department of Psychiatry and Mental Health, Faculty of Health Sciences, University of Cape Town, Anzio Road, Cape Town, South Africa; 2 Curtin enAble Institute, Curtin University, Kent Street, Bentley, WA Australia; 3 Mental Health, Alcohol, Substance Use, and Tobacco Research Unit, South African Medical Research Council, Parow, Cape Town, South Africa

**Keywords:** Tobacco use, Substance-related disorders, Smoking cessation, Health behaviour, Addiction treatment, South Africa, Low-and-middle-income country, Global South

## Abstract

**Introduction:**

Despite the high prevalence of tobacco use among people with substance use disorder (SUD), tobacco cessation is not a routine part of SUD treatment in South Africa. No studies have explored how people receiving SUD treatment view the effects of tobacco use on SUD treatment or their barriers to tobacco cessation. Clinicians need this information to develop locally relevant, person-centred tobacco cessation interventions. To address this gap, this study aimed to explore perceptions of tobacco use and its impact on SUD recovery among people receiving SUD treatment in South Africa.

**Methods:**

In-depth interviews were conducted with 20 participants (14 males, 6 females) recruited from five outpatient SUD treatment programmes in Cape Town, South Africa. On average, participants were 34.6 years old (SD = 10.1) and began smoking tobacco when 13.4 years old (SD = 2.6). At the time of the study, 40.0% (*n* = 8) had quit tobacco. Interviews explored experiences of tobacco use and its impact on SUD recovery. Data were analysed using reflexive thematic analysis.

**Results:**

The analysis generated four themes: (1) tobacco interconnects with other substance use; (2) psychosocial functions of tobacco: identity, connection and coping; (3) detrimental impact on SUD recovery; and (4) ambivalence about tobacco cessation, despite acknowledged benefits. Participants reported that their tobacco use intertwined with and reinforced their substance use, heightening cravings and impacting efforts to remain abstinent. Participants identified barriers to cessation that included using tobacco as a coping mechanism and as a tool for social connection, difficulties managing physiological withdrawal symptoms, and limited cessation support within SUD treatment and their social environments.

**Conclusion:**

Findings highlight the need for tobacco cessation interventions within South Africa’s SUD services. To overcome psychosocial and physiological barriers, SUD services should consider providing pharmacological interventions, psychosocial and behavioural counselling, and social support for tobacco cessation. Integrating these interventions into SUD treatment services could improve tobacco cessation rates and recovery outcomes for South Africans with SUDs.

**Supplementary Information:**

The online version contains supplementary material available at 10.1186/s13722-026-00660-8.

## Background

Globally, tobacco smoking remains a leading cause of premature death and health-related disability, accounting for 5.7% (4.7% − 6.8%) of disability-adjusted life-years and 9.1% (7.4%-10.6%) of premature deaths in 2021 [1]. Although the overall prevalence of tobacco use has declined significantly over the past three decades, these reductions have not been equitably distributed across populations [2]. The burden of tobacco-related harm has shifted to low-and middle-income countries (LMICs), which account for approximately 80% of the world’s 1.2 billion people who smoke tobacco [3, 4]. For example, South Africa (an upper-middle income country) has implemented extensive population-level tobacco control interventions [5] which initially led to a decline in tobacco smoking prevalence from a peak of 25.0% in 1998 to 19.4% in 2010 [6]. However, the trend has since plateaued with an estimated 20.2% of the adult population smoking tobacco in 2021 [7].

Disparities in tobacco use and associated harms are also more pronounced among persons with substance use disorders (SUD) [8]. Studies from high-income countries consistently show significantly higher tobacco smoking rates among persons with SUD compared to the general population [8–10]. While data from LMICs like South Africa remains limited, several studies report greater tobacco use among persons with co-occurring SUD [11–14]. This is a major concern given the synergistic effects of concurrent tobacco and other substance use on risk of chronic disease and early mortality [15–18]. Further, systematic reviews have shown that tobacco cessation during SUD treatment enhances both short and long-term recovery outcomes [19–21]. In contrast, individuals who continue to use tobacco are at greater risk of SUD recurrence, largely because tobacco activates the same neurobiological reward pathways as other substances and is often used together with other substances, prompting cravings for other substances even after extended periods of recovery [22–25]. These risks for SUD recovery are heightened for people who primarily smoke their illicit drugs [26].

Despite the high prevalence of tobacco use among persons in SUD treatment and evidence that cessation improves recovery outcomes [19, 27], there is inconsistent integration of tobacco cessation interventions into SUD treatment globally. Initial barriers to integrating tobacco cessation into SUD treatment included provider concerns that addressing smoking would deter individuals from seeking treatment and persistent misconceptions about tobacco cessation potentially undermining SUD recovery [28]. High-income countries (like the US and Australia) have made progress in integrating tobacco cessation into SUD treatment through implementing tobacco control policies and SUD provider training initiatives [29–32]. The voices of people who use SUD services have been integral to this progress by advocating for tobacco-free treatment environments and dispelling providers’ misconceptions that individuals receiving SUD treatment would not be receptive to tobacco cessation [33–36].

In contrast, the South African SUD treatment system has failed to address tobacco use amongst people with alcohol and drug use disorders despite the high prevalence of tobacco use in this population [37]. South Africa’s SUD treatment system is a mix of public and not-for-profit residential and outpatient services funded by the Department of Social Development. These services typically provide behavioural treatment for alcohol and other drugs and do not address tobacco use within the context of SUD treatment. Access to pharmacotherapy for alcohol, illicit drugs and tobacco cessation is limited to individuals who can afford to pay for these medications [38, 39]. While there have been initiatives to expand evidence-based intervention delivery and strengthen the SUD workforce [40–43], these efforts have not focused on integrating tobacco cessation interventions within SUD services. The absence of context-specific evidence on tobacco’s impact on recovery has contributed to this gap, with no prior studies examining South African perspectives on tobacco’s influence on SUD recovery. This gap is important to address as differences between South Africa and high-income countries may lead to divergent experiences with tobacco use and SUD treatment. Unlike settings where injection drug use predominates, the most frequently used illicit substances in South Africa —such as methamphetamine, cannabis, and methaqualone—are primarily smoked [14]. These drugs are sometimes mixed with tobacco to facilitate ingestion [44], potentially increasing the risk of SUD recurrence through shared behavioural and environmental cues. Additionally and in contrast to high-income countries [45, 46], pro-smoking social norms remain widespread in South Africa, particularly in socioeconomically disadvantaged communities [7, 47]. These norms may hinder tobacco cessation efforts for people with SUDs [48].

A deeper understanding of how people with SUDs in South Africa perceive the relationship between tobacco use and SUD treatment as well as their barriers to tobacco cessation is needed to inform the design of locally relevant, person-centred interventions [49]. In response, this study aimed to explore how South Africans with SUDs perceived the impact of tobacco use on SUD treatment and recovery.

## Methods

This qualitative study used a phenomenological design, framed within a constructivist epistemology, to examine how participants experienced and made meaning of their tobacco use in the context of SUD treatment and recovery [50]. The study is reported in accordance with COREQ guidelines.

### Study context

We recruited participants from intensive outpatient SUD treatment programs co-located within public primary care clinics operated by the City of Cape Town. These clinics operate in low-income communities characterised by high rates of poverty, HIV, crime, and SUD [51]. Persons presenting for treatment complete a comprehensive diagnostic assessment prior to program enrolment. Only individuals meeting diagnostic criteria for an alcohol or drug use disorder, as defined by the DSM-V, are eligible for treatment. Individuals who present with a tobacco use disorder without a co-occurring SUD are not eligible for this service. The SUD programs deliver the 16-week Matrix model that combines cognitive behaviour therapy, motivational interviewing, and contingency management with elements of 12-step facilitation. Weekly continuing care is offered to people who complete the initial program [43, 52]. These programs do not provide tobacco cessation services.

### Participants

Participants were recruited from five of eight possible Matrix sites. We excluded sites where the first author had been a senior clinician to minimise social desirability bias. At each site, senior clinicians used maximum variation sampling, a purposive sampling strategy designed to ensure broad representation of the study population [53], to identify people engaging with these services that could be referred to the research team for eligibility screening. Only two individuals identified through this process did not wish to be referred for study screening, both due to time constraints. The first author contacted consenting individuals by telephone to assess their eligibility. To be eligible, participants had to (i) be between 20 and 60 years old, (ii) have completed at least one month of treatment, (iii) self-report current or past tobacco (cigarette) use, and (iv) be comfortable communicating in English.

We used these procedures to recruit eight participants who had quit smoking during treatment and 12 who report current tobacco use (*n* = 20 in total, four per site). The concept of information power informed decisions about sample size, with 8–10 participants per group considered sufficient to generate rich findings when the research question is narrow in scope, criteria for participant inclusion are clearly defined, and interviews are conducted by experienced persons capable of facilitating high-quality dialogue [54].

### Procedures

After confirming eligibility, the first author (a female clinical social worker) obtained each participant’s written informed consent to be interviewed. While the first author had worked at Matrix sites, she had no prior relationship with study participants. A brief questionnaire was administered to all study participants prior to conducting the interviews. This questionnaire collected self-reported information about participants’ demographic characteristics (age, biological sex), tobacco use history (age of tobacco initiation, current tobacco use, intentions to quit) and substance use at treatment entry (primary substance for which treatment was sought, mode of use, duration of SUD recovery). This information was used to characterise the sample.

The first author, a female clinical social worker, conducted all interviews in English in private rooms at the Matrix sites. The interviewer used a semi-structured interview guide, developed with inputs from treatment providers and piloted with participants from another study [55] to facilitate the interviews. The interview guide (see Supplementary File 1) included open-ended questions and prompts about experiences of tobacco use, SUD treatment, and recovery. Interviews lasted between 60 and 90 min and were audio-recorded before being transcribed verbatim. Audio-recordings were supplemented with field notes and reflective memos made after each interview. We gave participants a gift for their time and offered referrals for tobacco cessation support. Participants were sent a copy of their interview transcript and invited to make changes. During the analytic process, participants were sent a summary of emergent themes and invited to provide feedback. The University of Cape Town’s Human Research Ethics committee (#152/2018) and the City of Cape Town’s Health Directorate Research Department approved the study.

### Data analysis

We used reflexive thematic analysis (RTA), following Braun and Clarke’s six-phase approach [56], to explore participants’ experiences of tobacco use and SUD treatment. We familiarised ourselves with the data by reviewing audio recordings and interview transcripts, documenting our initial observations and reflections through narrative memos [57]. Both authors independently coded all transcripts, meeting regularly to refine codes and reach consensus. We identified semantic and latent codes. No new codes emerged after coding twelve transcripts, suggesting inductive thematic saturation [58]. Following coding, we clustered codes with related content into preliminary themes and mapped evidence from each transcript to the corresponding theme. Member checking to review these emergent themes yielded no changes. We iteratively refined the themes through critical and reflective discussions before naming and defining the final set of themes and establishing connections between these themes to create an analytic narrative. NVivo 14 software was used for data management.

## Results

### Sample characteristics

Table 1 depicts the sociodemographic and clinical characteristics of study participants. Participants were 34.6 years old (SD = 10.1) on average and were predominantly male (70.0%, *n* = 14). Apart from tobacco, participants were not using other substances at the time of the interview, with their duration of SUD recovery ranging from one month to three years. On entering SUD treatment, all participants reported smoking drugs such as methamphetamine, methaqualone and cannabis. While most (*n* = 18, 90%) participants reported methamphetamine use, 60% (*n* = 12) of participants reported polysubstance use (defined as the use of two or more psychoactive substances other than tobacco). On average, participants began smoking tobacco when 13.4 years old (SD = 2.6). At the time of the study, 40.0% (*n* = 8) had ceased tobacco use. Among the 12 participants who reported current tobacco use, 11 (91.7%) expressed a desire to quit smoking.

**Table 1:** Demographic and clinical characteristics of the sample (*N* = 20)

Participant number		Sex	Age (years)	Age of tobacco initiation	Current tobacco use		Other Substance Use		Route of drug administration
1	Male		35	16	yes		Methamphetamine, Cocaine		Smoke and nasal
2	Male		29	13		no	Methamphetamine		Smoke
3	Male		51	15		no	Methamphetamine		smoke
4	Male		27	13		no	Methamphetamine		Smoke
5	Male		N/A*	12		yes	Methaqualone**		smoke
6	Male		N/A	16		no	Methaqualone		smoke
7	Male		N/A	13		no	Methamphetamine, Methaqualone, Cocaine, Heroin		Smoking, injecting, nasal
8	Male		N/A	16		yes	Methamphetamine		Smoking
9	Female		61	12		no	Methamphetamine		Smoking
10	Female		31	16		no	Methamphetamine, Cannabis		Smoking
11	Female		37	12		no	Methamphetamine, Alcohol		Smoking, oral ingestion
12	Male		36	8		yes	Methamphetamine		Smoking
13	Female		40	13		yes	Methamphetamine, Methaqualone, Cannabis		Smoking
14	Male		33	11		yes	Methamphetamine, Methaqualone, Cannabis		Smoking
15	Female		29	19		yes	Methamphetamine, Methaqualone, Alcohol		Smoking, oral ingestion
16	Male		23	16		yes	Methamphetamine, Methaqualone		Smoking
17	Female		34	13		yes	Methamphetamine, Methaqualone		Smoking
18	Male		39	12		yes	Methamphetamine, Methaqualone, Alcohol, Cannabis		Smoking, oral ingestion
19	Male		29	12		yes	Methamphetamine, Methaqualone, Alcohol, Cannabis		Smoking, oral ingestion
20	Female		20	10		yes	Methamphetamine, Methaqualone	Smoking	

*N/A (Not available) Ages not provided; ** Methaqualone (street name: Mandrax) is generally crushed, mixed with cannabis and tobacco and smoked in a pipe

### Themes

Four themes were generated from the data. The first theme, *Tobacco is interconnected with other substance use*, represents participants’ experiences of tobacco use and how it is intertwined with their substance use. The second theme, *Psychosocial functions of tobacco: identity, connection and coping*, reflects the idea that tobacco is integral to personal identity, informs social connections and supports psychological coping. Theme Three, *Detrimental impact on SUD recovery*, reflects participants’ views about the impact of continued tobacco use on SUD recovery. The fourth theme, *Ambivalent about tobacco cessation despite acknowledged benefits*, presents how participants acknowledged the benefits of cessation yet remained ambivalent about attempting change due to various barriers. These themes are described below and illustrated with participant quotes.

### Theme 1: Tobacco is interconnected with other substance use

Although participants had entered SUD treatment for methamphetamine, methaqualone, and cannabis-related difficulties, several people commented that tobacco was their primary substance of choice. Participants emphasised the significant role tobacco use played in their initiation and continued use of other substances. For instance, nearly all participants described how they had first started smoking tobacco before progressing to the use of other substances:

*I think the cigarette was the most important drug that I needed to have. And then obviously the other drugs came in. (Participant 15)*

*It was a gateway because it made it easier for me to start [using] other drugs. (Participant 19)*

For these participants, tobacco remained a constant feature in their lives. They described how they continued to smoke tobacco after they began using other substances and how their smoking persisted after they had stopped using these substances, attributing this persistence to tobacco’s addictive properties. Several participants described their desire to smoke tobacco as “overwhelming”, noting that these tobacco cravings often surpassed their desire for other drugs. As a result, some participants said they were willing to cease all other substance use, provided they could continue using tobacco:

*I could never put my cigarette aside for any other drug, for anything I ever used in my life. (Participant 9)*

*I started cigarettes, left weed [but] still do cigarettes, so the cigarettes is the one still continuing. (Participant 20)*

Several participants regarded their use of tobacco and other substances as mutually reinforcing and deeply intertwined. Many participants reported smoking cigarettes after consuming their primary substance to “extend the high”. Other participants described concurrent use, mixing tobacco with their drug of choice, believing this practice amplified and sustained the euphoric effects of the drug. Participant 13 reflected that

*It gave an awesome feeling. And I loved the feeling that I got from it. The high it actually gave me was better than just smoking my drug of choice alone.*

For some participants, the co-use of tobacco and other substances was so entrenched that they could not envision using one without the other. Participants described how smoking tobacco elicited cravings for other substances, and the use of other substances activated cravings for tobacco:

*I can’t have the one without the other. If I go buy my drug of choice, I make sure that I have cigarettes. (Participant 1)*

*And once I started smoking the drugs, I knew that I had to have cigarettes with everything. (Participant 15)*

Participants described how the interconnectedness between tobacco and other substance use resulted in their tobacco consumption increasing during periods of active substance use. They attributed increased tobacco consumption to both the concurrent use of tobacco and other substances and the use of tobacco as a mechanism for managing substance use withdrawal:

*When I actually started using Mandrax [methaqualone], then the usage of my cigarettes became more because now I need to mix cigarettes into the marijuana so that I can use my Mandrax. (Participant 13)*

### Theme 2: Psychosocial functions of tobacco: identity, connection and coping

Most participants reported initiating tobacco use during childhood. They described how early exposure to smoking within their family and social networks normalised tobacco use for them and resulted in them associating smoking with adulthood, maturity, and social belonging. This early exposure not only sparked curiosity about tobacco but also diminished any apprehension they had about smoking tobacco or other substances. As participants explained:

*If you play at others’ house and [you pretend] you’re a big lady you should have a cigarette in your hand because we used to watch everyone. All the adults had cigarettes in their hands… I guess it’s something (seen as) normal as an adult. (Participant 20)*

*What started it, I think, it’s because my mother was smoking, my father was smoking, my sisters was smoking and everybody around me was smoking. (Participant 9)*

Several participants reflected that initiating tobacco use during a critical stage of identity development contributed to their deep attachment to smoking. For some, tobacco use was so embedded in their sense of self that they could not imagine life without cigarettes. As participant nine commented:

*It became me, cigarettes became me…So maybe the cigarette will always stay a part of me subconsciously.*

In addition, many participants viewed smoking as a means of fostering social connections, believing that it facilitated entry into social groups. For these participants, smoking was a highly social activity. They described how they first started smoking with friends, emphasising the significance of sharing cigarettes within their social group which they thought strengthened friendships and enhanced feelings of belonging. As these participants articulated:

*So just to be social to be in. I guess that can be the reason why I just carried on smoking. (Participant 14)*

*Because if you don’t belong you don’t have anyone to cherish, or to share with…this is the reason why it’s easy for us to smoke because when you smoke, you share [cigarettes]. When you first smoke you don’t buy a cigarette, you are given by a friend. They teach you. That’s being loved in a way. (Participant 19)*

Some participants shared how their smoking evolved from a primarily social activity into a coping mechanism. The use of smoking as a strategy for coping with negative emotions was most evident amongst the third of participants who disclosed adverse childhood experiences or other traumatic experiences during the interview. Nonetheless, participants who did not disclose a history of trauma still viewed smoking as a tool for managing everyday stress. These participants were reluctant to quit smoking, citing a lack of alternative coping. As these participants commented:

*When I’m stressed. I’ll smoke a cigarette or two. Or when I’m worried, I’ll smoke a cigarette or when I’m having arguments with somebody and a bit worked up, I’ll have a cigarette to calm myself down. (Participant 14)*

*I would say a coping mechanism for stress. Emotions I can’t control. Because within recovery you get weird feeling sometimes and you don’t know what it is …. for me smoking is a coping mechanism for emotions and stress I can’t handle. (Participant 16)*

### Theme 3: Detrimental impact on SUD recovery

Although tobacco use was perceived to serve social and emotional functions, nearly all participants acknowledged its detrimental effects on physical health, emotional well-being. Many participants expressed concern that continuing to smoke tobacco would undermine and diminish their SUD recovery, with some describing it as a “dirty habit” and “another addiction” (Participant 2). Several participants stated that they could only consider themselves in recovery when abstinent from all substances, including tobacco — largely because they viewed tobacco use as the root of their other substance use difficulties. This view was shared by participants who had quit tobacco and by participants who continued to smoke tobacco. As these participants reflected:

*To me it seems like the cigarette can be actually the main dangerous aspect of my using if I can put it like that. (Participant 9)*

*They are the root of all evil. They are the root of it all. Cigarettes is where it [substance use] all began and then none of the others I believe would have followed. (Participant 14)*

Several participants described how tobacco use had negatively affected their own SUD recovery, ascribing these impacts to the interconnectedness of their tobacco and other substance use. These participants described how tobacco use in SUD recovery triggered flashbacks or vivid memories of past drug use, intensifying cravings for these drugs and making it harder to sustain abstinence. They thought these experiences were a consequence of both tobacco and other substances sharing an administration route (smoking) and using similar paraphernalia such as lighters, matches, and pipes. For some, even the sight or smell of cigarette smoke or handling of cigarettes and lighters evoked strong associations with their former substance use and urges to use these substances:

*I smoke my cigarettes. Flashbacks would come, of me like smoking when I used to use and that stuff. So that would come up… And it’s almost like if you smoke a cigarette you going to use again. (Participant 10)*

*I would smoke a cigarette, and I would blow out and then my mind would go [to the other drugs] and that’s it. Like the smoke reminds me of the meth smoke… I did it for a while and I went back to smoking [meth]. I relapsed. (Participant 8)*

Due to the perceived impact of tobacco use on substance use craving, some participants attributed prior difficulties in maintaining SUD recovery to their continued use of tobacco. This belief was mainly held by participants who had stopped smoking, with several describing how they had quit tobacco to sustain their SUD recovery:

*It can maybe take you back to these drugs. That’s what was happening to me. That’s what I was getting, the message through my situation. I had to do [quit] both, because smoking almost always triggered me going back to the drugs, so it’s going to be very dangerous for me to keep on smoking. (Participant 2)*

*Because cigarettes led me back to smoking tik [methamphetamine]. And cigarettes lead me to smoking of buttons [Mandrax] in the pipe, and the weed again. (Participant 7)*

### Theme 4: Ambivalence about tobacco cessation despite acknowledged benefits

Participants who had successfully quit smoking emphasised the physical and mental health benefits of tobacco cessation. They also described how tobacco cessation enhanced their self-esteem by enabling them to construct a substance-free identity and reconnect with the version of themselves that existed prior to tobacco and other substance use. They reflected on the sense of accomplishment gained through cessation, which further enhanced their self-esteem. As these participants explained:

*Deciding to give up cigarettes with my using was because I decided to become a whole new person. I decided to become the person that I knew I always was before I started using a cigarette you know. (Participant 9)*

*I’ve got so much self-worth. I really love myself now being clean and sober and not using drugs. I’m not all together yet, but I have a tremendous amount of confidence now. (Participant 3)*

Participants who were using tobacco also acknowledged the potential health benefits of quitting tobacco, with many expressing a desire to quit smoking. Yet most of the participants who were still using tobacco seemed ambivalent about committing to change their tobacco use. For instance, some initially asserted that quitting tobacco would be easy, but later in the same interview acknowledged that tobacco cessation was difficult, as expressed by Participant 17:

*Over the years I’ve been able to stop for a few months and start again. It’s not a drug really, I wouldn’t classify cigarettes as something difficult to get over. It’s not. I guess the nicotine has its power over you… It’s not a problem. I would say I smoke a hell of a lot more since I stopped smoking drugs, but again I can stop easily.*

Several psychosocial barriers seemed to contribute to this ambivalence among people who were using tobacco. For many participants using tobacco, this ambivalence stemmed from concerns about losing a key mechanism for coping with stress and negative emotions. Several participants who were still smoking worried about being able to manage stressful situations, emotional distress, and anxiety without tobacco. Consequently, these participants perceived tobacco cessation as too great a challenge when undertaken alongside SUD treatment. As Participant 14 reflected:

*And naturally I know that if I stop smoking cigarettes today, I’m going to live so much longer. But I don’t want to take up too many challenges right now. I feel that personally I feel that I can do it. I just choose not to right now.*

For other participants who were still smoking, multiple failed cessation attempts had eroded their confidence to sustain tobacco cessation. Almost half of the participants who were ambivalent about making changes to their tobacco use believed they lacked the necessary skills and supports to quit smoking. As Participant 15 said, *“But my cigarettes I don’t think I can leave it. I want to, but I don’t think I can.”* These participants described how previous unsuccessful quit attempts had left them feeling discouraged and unmotivated. Although some participants had tried to use the behavioural strategies learned during SUD treatment to support tobacco cessation, these strategies did not help them manage nicotine withdrawal. As these participants explained:

*I know for a fact it’s more difficult to give up smoking cigarettes than to give up drugs. I’m so sure. Drugs I gave up very easily. Cigarettes not so much. (Participant 1)*

*It [quit attempt] was unsuccessful. Because as hard as I try, I’m still a bit too weak for that. My craving for the cigarette is still overpowering my thoughts of not wanting to light that cigarette. (Participant 12)*

Additionally, a small number of participants who were using tobacco expressed uncertainty about quitting tobacco because they believed that smoking helped them to maintain their SUD recovery. These participants explained that they used tobacco as a replacement for other substances and to manage cravings. They expressed concern that quitting tobacco may jeopardise their recovery. As these participants explained:

*I’m substituting, because obviously at the back of my mind I’m getting this little drug craving, but me myself I’m not craving it. But I think my sub-conscience is substituting it with smoking cigarettes. (Participant 17)*

*That’s actually the one problem I’m scared of. If I give up smoking entirely, give up cigarettes, I’m scared I’m going to slip up you know. (Participant 1)*

Almost all participants, irrespective of current smoking status, described how they received little information on the benefits of tobacco cessation for SUD recovery or resources to support cessation while in SUD treatment. Consequently, some participants felt their efforts to quit smoking were unsupported by their SUD provider. For instance, Participant 6 shared that their provider did not encourage them to quit smoking tobacco, viewing it as less of a priority than other substances:

*They [treatment providers] said to me cigarettes is not a drug. They don’t see it as a drug. But it is a drug to me. How could you say such a thing. If you smoke cigarettes, I am telling you it will remind you of your other drugs.*

## Discussion

This study is the first to explore how people receiving SUD treatment in South Africa view the relationship between tobacco use and SUD recovery and barriers to tobacco cessation. Contrary to perceptions that persons with SUDs may not be interested in tobacco cessation, we found that South Africans in SUD recovery—like their counterparts from high-income countries [33–36] — largely recognise the impact of tobacco use on SUD recovery and are motivated to quit smoking to support their SUD recovery. In this study, most participants recognised that their tobacco use was closely linked to other drug use, elicited drug cravings, and contributed to the resumption of drug use after periods of SUD recovery. While many participants had attempted to stop smoking, even those who had successfully stopped smoking reported that this required multiple quit attempts due to physiological and psychosocial barriers to quitting and minimal support for tobacco cessation during SUD treatment.

In this study, the use of tobacco as a mechanism for coping with psychosocial and emotional stress emerged as a key cessation barrier for people who were using tobacco during SUD treatment. This is consistent with results from a systematic review of barriers to smoking cessation among people with SUDs [59]. Like prior South African studies that documented the widespread use of alcohol, tobacco, and other drugs as a strategy for coping with stress [60–62], study participants described using tobacco to manage stress and regulate their emotions. They were reluctant to quit smoking because they feared that giving up this coping strategy might jeopardise their SUD recovery. This finding suggests that interventions that enhance skills for coping with stressors may yield benefits for tobacco cessation. Brief problem-solving therapy (PST) is an example of such an intervention. This intervention has been tailored for South Africans with SUDs [62], with trials demonstrating significant improvements in coping skills and SUD and mental health outcomes [63–65]. In addition, several participants who were still smoking indicated they lacked the behavioural skills required to successfully quit smoking. As such, offering a combination of evidence-based behavioural counselling for tobacco cessation [66] and coping interventions like PST to people with SUDS may result in better tobacco cessation and SUD recovery outcomes.

Our findings also illustrate how the social rewards associated with tobacco use impede cessation efforts. Participants shared how socio-cultural norms that supported and encouraged tobacco use, social modelling, and community-level factors contributed to early tobacco use and its entrenchment through powerful social rewards. Many participants who were still smoking were concerned about that tobacco cessation would lead to the loss of these social rewards. Consistent with prior evidence that pro-smoking environments hinder tobacco cessation [62, 65], participants described how pro-smoking norms within their communities and the absence of tobacco cessation support from their social networks undermined their cessation efforts. Prior South African studies have identified similar community-level barriers to SUD treatment and recovery [67, 68]. SUD treatment programs offer a unique opportunity to counter these well-established social and contextual barriers to tobacco cessation [59]. Peer-led recovery support groups are a part of many South African SUD treatment services, including the Matrix program [43], with evidence of the feasibility, acceptability, and effectiveness of these interventions for mental health and SUD recovery [69, 70]. With modest investment, these groups could be supported to provide tobacco education practical behavioural and coping skills training to support cessation, and social encouragement for tobacco cessation. While these peer-led interventions have been shown to improve tobacco cessation outcomes in high-income countries [71], they have yet to be tested in LMICs like South Africa.

Further, study findings indicate that the physiological effects of tobacco are a major barrier to cessation. Participants reported long histories of heavy consumption and multiple unsuccessful quit attempts, largely due to difficulties managing tobacco withdrawal and cravings —symptoms indicative of severe tobacco use disorder. To overcome these physiological barriers to tobacco cessation, people in SUD treatment require access to evidence-based pharmacotherapy for tobacco use disorder such as nicotine replacement therapy, bupropion, or varenicline [72]. Research shows that combining these pharmacotherapies with behavioural interventions significantly increases the likelihood of successful cessation among people in SUD treatment compared to either approach alone [59, 73, 74]. Yet access to these pharmacotherapies is restricted to South Africans who can afford to purchase them privately [75], creating barriers for individuals in disadvantaged communities who use public health services. Given the high co-occurrence of tobacco use and SUD—and evidence that smoking can intensify drug cravings and hinder recovery [22, 23]—expanding access to these pharmacotherapies should be a public health priority. State-funded provision of nicotine replacement therapy and other pharmacotherapies for TUD would likely improve tobacco cessation rates as well as SUD treatment outcomes.

Although this study identified several barriers to tobacco cessation among people in South African SUD services, there are several limitations warrant further consideration. The study sample is relatively small and selected from outpatient SUD programmes in Cape Town that deliver the same model of care. While the age, gender, and substance use profiles of study participants appear representative of people entering SUD treatment in this setting [37], we acknowledge that findings may not be transferable to individuals from communities with less tobacco use, or people engaged in different forms of SUD treatment. Additionally, all participants were socio-economically disadvantaged which likely influenced their access to cessation resources and shaped their experiences and perceptions of quitting. Selection bias is also possible as therapists may have approached persons who they considered suitable for study participation, potentially influenced by their own views on tobacco use and recovery. Additionally, as this study focused on the perspectives of people in recovery, we do not know how treatment providers viewed tobacco use or the provision of tobacco cessation supports during SUD treatment, and whether their views differed from those of people receiving SUD treatment. Further research that examines providers’ perspectives on the need for and potential benefits of supporting tobacco cessation during SUD treatment could strengthen the rationale for investing in these additional services.

Despite these limitations, our findings hold implications for South African SUD treatment policy and practice, highlighting the opportunity to strengthen SUD recovery outcomes through integrating tobacco cessation interventions as part of routine SUD treatment. Given our findings of physiological and psychosocial barriers to tobacco cessation, SUD treatment services should consider providing a combination of pharmacotherapy, coping skills and social support for tobacco cessation. We recognise that implementing tobacco cessation interventions will represent a major practice change for South African treatment services, and there may be patient-, provider, and system-level barriers to their adoption. For example, some participants reported that their treatment providers had dismissed their concerns about their continued tobacco use, raising questions about treatment providers’ attitudes towards tobacco cessation during SUD treatment. As attitudes towards tobacco can influence provider willingness to deliver cessation interventions [35], this barrier will be critical to address in order to ensure successful integration of tobacco cessation supports within SUD programs. Further research is needed to systematically identify patient-, provider, and system-level barriers to the implementation of these interventions and to develop strategies for facilitating this practice change.

## Conclusion

Our findings highlight how tobacco use negatively affects SUD recovery, underscoring the importance of providing tobacco cessation interventions as part of SUD treatment. In this study, individuals with SUDs were motivated to stop smoking but faced psychosocial, and physiological barriers to cessation that had not been addressed during SUD treatment. To overcome these barriers, people receiving SUD treatment may require a combination of pharmacological interventions, psychosocial and behavioural counselling, and social support for tobacco cessation. Successful implementation of these tobacco cessation supports will require treatment providers to be trained to effectively deliver these interventions, program funding to be expanded to include the costs of delivering these new services, and policy reform to enable access to pharmacological interventions for tobacco cessation via South Africa’s public health system. While implementing tobacco cessation interventions will likely support better health and recovery outcomes for South Africans with SUDs, these interventions could have greater impact when complemented by population-level tobacco control strategies and interventions to shift pro-smoking social norms.

## Supplementary Information

Below is the link to the electronic supplementary material.

Supplementary Material 1

## Data Availability

De-identified data cannot be shared publicly as participants did not consent to the sharing of their data. Study materials are available on reasonable request from the corresponding author.
