# Supplementary material for: “Cigarettes led me back to smoking tik”: lived experience perspectives on tobacco use during substance use treatment and recovery in South Africa
Source: Addict Sci Clin Pract. 2026 Mar 12;21:29. doi: 10.1186/s13722-026-00660-8 (PMC13001285; doi:10.1186/s13722-026-00660-8)
Supplement: Supplementary file 1 — Supplementary Material 1 [file 13722_2026_660_MOESM1_ESM.docx]

**Supplementary File 1: Semi-Structured Interview Guide**

**Introduction Script**

Hello, my name is _________and I am conducting research on recovery and how the use of cigarettes affects this process. I am going to ask you some questions about your recovery and the use of cigarettes in order to better understand the relationship that may exist between these. This interview is going to take about an hour to an hour and a half. Are you available to answer some questions?

**Interview Questions**

**1. Cigarette Use History**

**First, let's start by talking about your use of cigarettes. Can you give me an overview of your cigarette use?**

**Probes:**

- When did you start smoking cigarettes?
- How did you start smoking cigarettes?
- Why did you start smoking?
- How did smoking make you feel?
- How did smoking cigarettes after a while make you feel regarding the following?
  - Yourself
  - Other drugs?
- What are some of the effects of smoking cigarettes on your physical, social, mental self?

**2. Other Drug Use History**

**Next, I would like to explore your use of other drugs. Please tell me the story of your drug use.**

**Probes:**

- When did you start using other drugs—what did you use, how long and what method did you use for your drug of choice?
- To what extent did your cigarette use change when you started using other drugs?

**3. Relationship Between Cigarette and Other Drug Use**

**For you, what was the relationship between cigarettes use and other drugs?**

**Probes:**

- How do you feel after you have smoked drugs?
- Did you use cigarettes with other drugs or alcohol?
- How was using/smoking cigarettes linked to drug use for you?
- When did you use cigarettes—before or after or during your drug/alcohol use?
- How does smoking cigarettes when you are using drugs and/or alcohol make you feel?
- How did using alcohol or drugs change the amount of cigarettes you were smoking?

**4. Cigarettes and Recovery**

**In your view, how did cigarette use help or hinder your recovery?**

**Probes:**

- To what extent did smoking cigarettes change when you entered treatment? Did you want it to change?
- In recovery, how does smoking cigarettes affect you?
- What role did smoking cigarettes have in your recovery?
- What are some of the strategies you have used to make changes to your cigarette use in recovery?

**Closing**

We have come to the end of the interview. Is there anything else you would like to tell me about smoking cigarettes and recovery from alcohol or drug use?

Thank you so much for your willingness to participate in this interview. Your participation has been invaluable.
